# Evaluation of Two Different Analytical Methods for Circulating Tumor Cell Detection in Peripheral Blood of Patients with Primary Breast Cancer

**DOI:** 10.1155/2014/491459

**Published:** 2014-04-08

**Authors:** B. A. S. Jaeger, J. Jueckstock, U. Andergassen, J. Salmen, F. Schochter, V. Fink, M. Alunni-Fabbroni, M. Rezai, Th. Beck, M. W. Beckmann, K. Friese, T. W. P. Friedl, W. Janni, B. Rack

**Affiliations:** ^1^Department of Gynecology and Obstetrics, University Hospital Ulm, 89075 Ulm, Germany; ^2^Department of Gynecology and Obstetrics, University Hospital Munich, Ludwig-Maximilians-University, 80337 Munich, Germany; ^3^Luisen Hospital, 40235 Duesseldorf, Germany; ^4^RoMed Hospital Rosenheim, 83022 Rosenheim, Germany; ^5^Department of Gynecology and Obstetrics, University Hospital Erlangen, Friedrich-Alexander University Erlangen-Nuremberg, Comprehensive Cancer Center Erlangen-EMN, 91054 Erlangen, Germany

## Abstract

*Background*. Evidence is accumulating that circulating tumor cells (CTC) out of peripheral blood can serve as prognostic marker not only in metastatic but also in early breast cancer (BC). Various methods are available to detect CTC. Comparisons between the different techniques, however, are rare. * Material and Methods*. We evaluate two different methods for CTC enrichment and detection in primary BC patients: the FDA-approved CellSearch System (CSS; Veridex, Warren, USA) and a manual immunocytochemistry (MICC). The cut-off value for positivity was ≥1 CTC. * Results*. The two different nonoverlapping patient cohorts evaluated with one or the other method were well balanced regarding common clinical parameters. Before adjuvant CHT 21.1% (416 out of 1972) and 20.6% (247 out of 1198) of the patients were CTC-positive, while after CHT 22.5% (359 out of 1598) and 16.6% (177 out of 1066) of the patients were CTC-positive using CSS or MICC, respectively. CTC positivity rate before CHT was thus similar and not significantly different (*P* = 0.749), while CTC positivity rate immediately after CHT was significantly lower using MICC compared to CSS (*P* < 0.001). * Conclusion*. Using CSS or MICC for CTC detection, we found comparable prevalence of CTC before but not after adjuvant CHT.

## 1. Introduction

There is consistent data showing the prognostic relevance of CTC in metastatic BC. A CTC count of ≥5 CTCs per 7.5 mL was significantly associated with shortened overall survival (OS) and progression free survival (PFS) [1–4]. However, there is limited data on the prognostic value of CTC in early BC. Using the semi-automated detection method CSS (Veridex, Warren, USA), we could show that the count of CTC at the time of first diagnosis of an operable disease has an influence on OS and PFS of patients with BC [5]. Recent data proofed that CTC positivity predicted both decreased PFS and OS in early BC [6].

CTC with epithelial characteristics are a rare event in the peripheral blood of cancer patients both in terms of absolute numbers (<10 cells/mL) and in terms of relative numbers as compared to other blood cells (one CTC per 10^6^–10^7^ leukocytes) [7]. Various methods for isolation and characterization of CTC are available that differ with regard to enrichment, staining and detection [8] as well as sensitivity, specificity and reproducibility [9]. Currently used techniques rely on a first cellular enrichment step to isolate CTC from other cell types such as red blood cells and leukocytes. Physical (filters and density gradients) and immunomagnetic approaches (magnetic affinity cell sorting, magnetic beads and ferrofluid-based systems) are common examples of cell enrichment methods [10]. Identification of CTC is based on either direct cytometric methods using antibodies such as immunocytochemistry (ICC), immunofluorescence (IF), or flow cytometry (FACS), or indirect nucleic acid-based methods which measure mRNA transcripts by reverse transcriptase-polymerase chain reaction (RT-PCR) [9, 10].

Further technologies aim at higher sensitivity and better phenotyping of CTC. Therefor evaluation and comparison of different approaches is needed. CSS is a semi-automated detection method based on an immunomagnetic enrichment using magnetic microbeads directed against the epithelial marker EpCAM. This step is followed by an IF staining for cytokeratin CK (CK8, CK18, CK19) and CD45 to distinguish epithelial cells from leukocytes. A staining for nucleus acid dye detects vital cells. After these automated preparation steps the detection of CTC is done visually. Until today it is the only CTC detection method which has been cleared by the U.S. Food and Drug Administration for use in patient care [11, 12].

Using MICC for CTC detection the enrichment is based on a density gradient centrifugation as in our study OncoQuick (Greiner BioOne, Frickenhausen, Germany), which separates mononuclear cells from leukocytes and erythrocytes. It consists out of tubes with a porous barrier and a separation medium. It is efficient for the enrichment of CTC in the whole blood. A total of 1 × 10^6^ mononuclear cells are poured on a microscopic slide which is air dried. Subsequently cells are stained for CK (CK8, CK18, CK19). No staining for the nucleus or for CD45 is performed. CTC are detected by conventional light microscopy.

In this study we detected CTC in a large patient cohort with early BC before and after adjuvant CHT treated in the SUCCESS A trial [13]. The aim of this analysis was to evaluate CTC positivity rate and CTC load using two different detection methods (CSS and MICC) in two different not overlapping but comparable patient groups in order to establish these methods for further patient care.

## 2. Patients, Material and Methods

### 2.1. Patients

Our patients' collective was treated within the SUCCESS A trial and defined as women with histologically confirmed, invasive primary BC. All patients had either node positive or high-risk node negative disease. Mastectomy or breast conservation leading to R0 resection in all cases was performed as primary surgery. 3754 patients were enrolled in this German trial in a time period from 2005 to 2007. All enrolled patients gave their informed written consent for study inclusion and the research project. As a translational research project CTC were assessed before and right after CHT. The SUCCESS A trial is a multicenter, randomized phase III study, which compared patients treated with 3 cycles of 5-fluorouracil, epirubicin and cyclophosphamide (FEC) followed by 3 cycles of docetaxel (D) every three weeks (q3w) versus 3 cycles of FEC followed by 3 cycles of gemcitabine (G) and docetaxel (D) q3w as CHT. Parameters analyzed at primary diagnosis were the following: age, tumor stage, nodal stage, histological grading, histological type, estrogen (ER) and progesterone receptor (PR) status, HER2 status and menopausal status.

**Figure 1:**
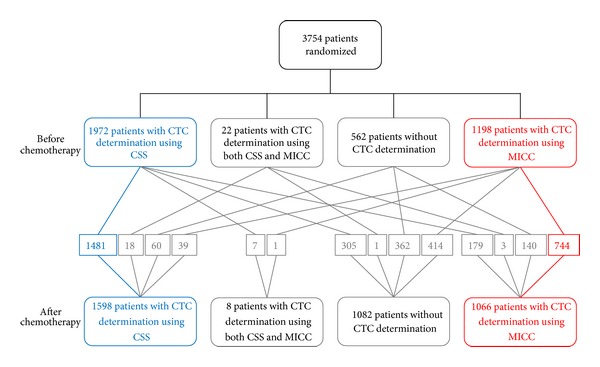
Flow chart illustrating the patient selection process.

Two different methods to detect CTC were prospectively evaluated in two not overlapping but comparable patient cohorts of the whole study population. Due to lack of unlimited blood volume, samples were assigned to one or the other method. Out of the 3754 patients randomized for the clinical treatment study CTC detection was conducted in 3170 patients before CHT and in 2664 patients after CHT. Which method was used for CTC detection was prearranged: CSS was conducted for the first patients recruited (2000 patients planned). If CSS was not available (e.g., due to technical issues) and for the patients recruited later in the course of the clinical trial MICC was conducted for CTC detection. The patient selection process is illustrated in Figure 1. The corresponding blood sample of one patient after CHT was planned to be analyzed with the same method used for the sample before CHT (blue and red cohort in Figure 1). In order not to compromise statistical independency of the two groups to be compared (blood samples analyzed for the presence of CTC using either CSS or MICC), patients for whom CTC presence was investigated using both methods simultaneously were excluded from the analyses (22 cases before chemotherapy, 8 cases after chemotherapy).

Both methods were used according to the manufactures' instructions with minor modifications (as described below). The trial and the examination of blood samples were approved by the local ethic committees and conducted in accordance with the Declaration of Helsinki.

### 2.2. CTC Detection Using the CellSearch System

A total of 3570 samples (1972 samples before and 1598 after CHT) were analyzed using the CSS (Veridex, Warren, USA), which consists of the CellTracks AutoPrep System and the CellTracks Analyzer II. Prior to any therapy and right after CHT, about 30 mL of blood were collected into CellSave blood collection tubes (Immunicon, Inc., Huntingdon Valley, PA, USA) at the local site during routine blood draw by peripheral vain puncture. These tubes are evacuated blood drawtubes containing EDTA and a cellular preservative not described in details by the supplier. The CellSave tubes were used to maintain cell integrity and avoid cell degradation. The samples were then sent to the laboratory for tumor immunology at the Department of Obstetrics and Gynecology, Klinikum Innenstadt of the Ludwig-Maximilians-University (LMU), Munich, Germany for further investigation. There they were examined within a maximum of 96 h after blood drawing, which is the time period for which the vendor guarantees valid results after processing [14].

The samples were run with the Epithelial Cell Kit (Veridex, Warren, USA) and the CellTracks AutoPrep System as described before [15]. In brief, this system is based on immunomagnetic enrichment with an EpCAM-antibody, followed by labeling with monoclonal antibodies specific for CK (CK8, CK18, CK19) and leukocytes (CD45). For that, the CellSearch Epithelial Cell kit contains all required reagents to conduct these steps: the ferrofluid particles coated with anti-EpCAM antibodies for the immunomagnetic enrichment, two phycoerythrin-conjugated antibodies directed against CK to specifically identify epithelial cells, a permeabilization buffer to allow CK antibodies entry into epithelial cells, a nuclear dye [4,6-diamidino-2-phenylindole (DAPI)] to fluorescently label the cell nuclei and an antibody against CD45 conjugated with allophycocyanin to identify leucocytes.

For the sample processing and evaluation the blood contained in three CellSave tubes were pooled using a ficoll density gradient. Then 7.5 mL of enriched blood were gently mixed with 6.5 mL of dilution buffer, centrifuged for 10 min at 800 g at room temperature, and transferred into the CellTracks AutoPrep System. The instrument does all remaining steps automatically. The final volume of 300 *μ*L containing enriched CTC is then transferred automatically to a cartridge in a MagNest (magnetic device for incubation) and is placed inside the MagNest cell presentation chamber. After an incubation time of 20 min to a maximum of 24 h in the dark at room temperature, evaluation of the samples was done using the CellTracks Analyzer II, a semiautomated four-color fluorescence microscope. The captured images are presented in a picture gallery on a computer screen. Two independent readers classified the cells according to the following criteria: First, the staining patterns must be consistent with that of an epithelial cell (cytokeratinphycoerythrin positive/DAPI positive/CD45-allophycocyanin negative). Second, it has to be nearly round or oval with a visible nucleus within the cytoplasm. Third, CTC must have a minimum size of 4 *μ*m, however present with a large heterogeneity regarding both CTC size and morphology [14].

### 2.3. CTC Detection Using a Manual Immunocytochemistry

The total number of samples analyzed using the MICC was 2264 (1198 samples before and 1066 samples after CHT). CTC were isolated from an EDTA evacuated blood draw tube and when available from the CellSave tube (total of 40 mL blood). The tumor cell enrichment was done by a density gradient centrifugation with the OncoQuick (Greiner BioOne, Frickenhausen, Germany) system. Compared to Ficoll-Hypaque (density of 1.077 g/mL), it uses a liquid separation medium optimized for the specific enrichment of CTC and an additional membrane. Density gradient centrifugation separates CTC and mononuclear cells from blood cells and granulocytes. However, CTC can easily be lost using this technique due to the presence of aggregates or to the migration of cells to the plasma layer. OncoQuick however reduces the cross-contamination of the different layers [10]. Cell separation was performed according to manufacturer's protocol [16]. In brief, precooled 50 mL OncoQuick vials were overlaid with the blood and centrifuged continuously for 25 min at 1105 g and 6°C. The entire volume of the compartment with interphase cells was poured into a fresh centrifugation tube. Cells were centrifuged twice with washing buffer (1 L PBS and 5 g of bovine albumin) at 209 g for 10 min at 6°C without break. If necessary red blood cells were lysed with lysis buffer (155 mM NH4Cl, 10 mM KHC03, 0.1 mM EDTA pH 7.2) according to the recommendations of the supplier R & D systems. 1 × 10^6^ mononuclear cells were spun onto a glass slide (cytospin), using a cytocentrifuge (Hettich, Tuttlingen, Germany) [16]. 2 out of a maximum of 6 cytospins as well as one negative control (see below) were prepared from each blood sample. In addition to be sure that the staining worked, each time a positive control with cytospins containing MCF7 or SKBR3 cells (as available) were prepared. The slides were left to air-dry overnight (12 to 24 h) at room temperature [17], followed by staining or storage at −80°C.

CTC detection was done by MICC, which is an open system with respect to the selected antibodies. Staining was performed using the anti-CK antibody A45-B/B3 (Micromet, Munich, Germany), which recognizes CK8, CK18, and CK19 [18–20]. For the detection of CTC, an ICC staining based on the alkaline phosphatase-antialkaline phosphatase (APAAP) technique was performed using the Z0259 antibody (Dako) as secondary antibody. Levamisole was taken for blocking endogenous alkaline phosphatase [16]. For the negative control the staining was done with the MOPC-21 antibody (Mouse IgG1, k; Sigma) instead of A45-B/B3 and the secondary antibody. Afterwards the slides were sealed with coverslips and stored at room temperature. Conventional light field microscopy (Axiophot; Zeiss, Oberkochen, Germany) was used for the detection of stained cells. The slides were analyzed by two independent observers. CTC were defined as bright red, round or oval shaped events with a minimum of 4 *μ*m in size.

**Figure 2:**
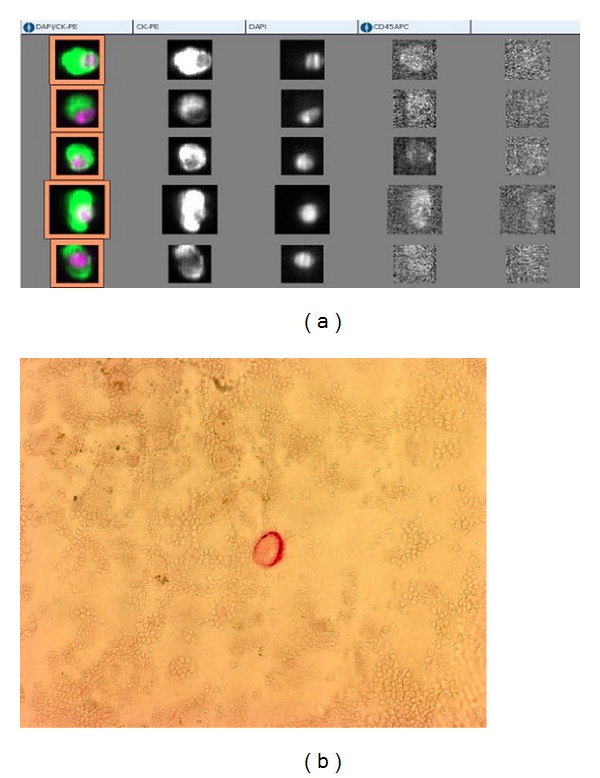
(a) shows images with CTC and artifacts from one patient presented in the gallery of the CSS. CTC show the characteristics defined before: round or oval shape, positive signal in the cytokeratin channel (second column), intact nucleus (third column), overlapping of nucleus and cytokeratin signal (first column), as well as no signal for CD45 as leucocytes marker (column 4), and in the negative control channel (fifth column). In comparison (b) shows a sample of a CTC detected by the MICC. Cells which are labeled with the anti-cytokeratin-antibody A45-B/B3 are then detected by the Z0259 antibody using the APAAP method and appear bright red.

For both methods, the cut-off value for positivity was ≥1 CTC.  Figure 2 shows sample pictures of both detection methods.

### 2.4. Statistical Analyses

All statistical analyses were conducted using the program IBM SPSS Statistics (Version 19). *P*-values below 0.05 were considered statistically significant. Descriptive statistics for all categorical data are summarized using frequency tables presenting absolute and relative frequencies. All tests regarding comparisons of patient or tumor characteristics between groups, associations between patient or tumor characteristics and the prevalence of CTC, or comparisons of CTC prevalence between methods were conducted using the Chi-Square test for all categorical data. Comparisons between groups regarding patient age were performed with the Mann-Whitney-*U* test.

## 3. Results

### 3.1. CTC Prevalence before Chemotherapy

Before CHT 1972 samples were analyzed using CSS and 1198 using MICC, in total 3170. The two patient cohorts were well balanced with respect to the common baseline patient and clinical parameters, which were listed in Table 1 (all *P* > 0.15). The majority of the patients showed pT1 or pT2 tumor stage, pN0 or pN1 nodal status and was postmenopausal. Concerning the histology most of the patients had a G2 or G3 histological grading and a ductal invasive BC, a positive ER and PR status as well as a negative HER2 status.

CTC positivity as assessed using CSS (21.1%) was significantly associated with positive lymph node status (*P* < 0.001), but not with any other of the clinico-pathological variables listed in Table 1 (all *P* > 0.05). There was no significant association between CTC positivity as assessed by MICC and any of the clinico-pathological variables (all *P* > 0.2).

**Figure 3:**
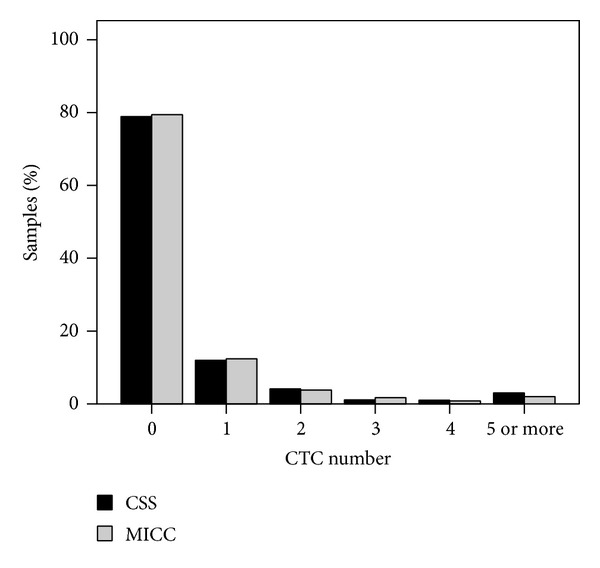
Distribution of the number of CTC detected before chemotherapy using the CellSearch System (CSS, black bars; *n* = 1972) or manual immunocytochemistry (MICC, gray bars; *n* = 1198).

In 1556 (78.9%) of the 1972 samples analyzed for the presence of CTC before CHT using CSS, no CTC were detected, while 416 (21.1%) were positive for CTC (median 1 CTC, range 1–827 CTC). 236 (12.0%) of the samples showed 1 CTC, and higher CTC loads were found in less than 10% of all samples with 80 (4.1%), 21 (1.1%) and 19 (1.0%) of the samples containing 2, 3 and 4 CTC respectively, while 5 CTC or more were detected in only 60 (3.0%) of the samples (Figure 3).

Out of the 1198 samples investigated for the presence of CTC before chemotherapy using MICC method, 951 (79.4%) were negative for CTC. In the majority of the 247 (20.6%) positive samples (median 1 CTC, range 1–256 CTC) only 1 CTC was detected (*n* = 148, 12.4%). Higher numbers occurred in less than 9% of all samples, with 45 (3.8%), 20 (1.7%), 10 (0.8%) and 24 (2.0%) of the samples containing 2, 3, 4, and 5 or more CTC, respectively (Figure 3).

The CTC positivity rate before CHT as assessed by the two methods did not differ significantly (CSS: 21.1% versus MICC: 20.6%, *P* = 0.749). The distributions of CTC numbers as detected before CHT using CSS or MICC are shown in Figure 3. The two distributions were very similar and not significantly different (*P* = 0.351).

### 3.2. CTC Prevalence after Chemotherapy

A total of 2664 blood samples were analyzed for the presence of CTC after adjuvant CHT, with 1598 samples being investigated using CSS and 1066 samples being investigated using MICC. Again, the two groups were well balanced with respect to the common baseline patient and clinical parameters, which are listed in Table 2 (all *P* > 0.05).

**Figure 4:**
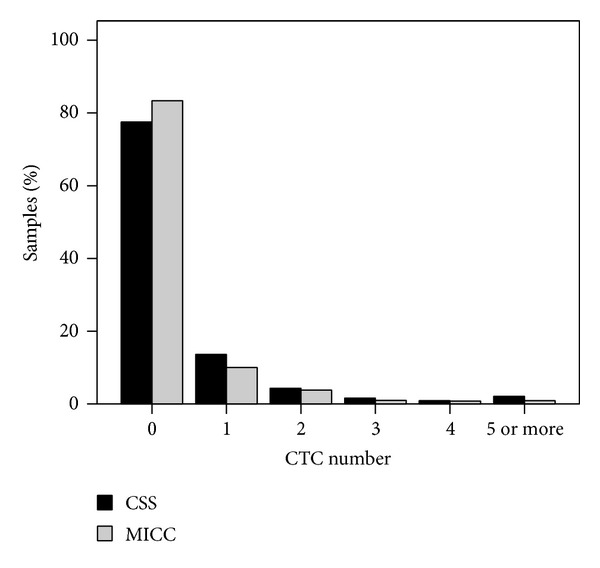
Distribution of the number of CTC detected after chemotherapy using the CellSearch System (CSS, black bars; *n* = 1598) or manual immunocytochemistry (MICC, gray bars; *n* = 1066).

No CTC were found in 1239 (77.5%) of the 1598 samples analyzed for the presence of CTC after CHT using the CSS. Of the 359 (22.5%) CTC positive samples (median 1 CTC, range 1–124 CTC), 217 (13.6%) samples had 1 CTC, 68 (4.3%) had 2 CTC, 26 (1.6%) had 3 CTC, 14 (0.9%) had 4 CTC, and 34 (2.1%) had 5 or more CTC (Figure 4).

889 (83.4%) of the 1066 samples investigated for the presence of CTC after CHT using MICC were negative for CTC and CTC were found in the remaining 177 (16.6%) samples (median 1 CTC, range 1–23 CTC). One CTC was detected in 107 (10.0%) samples, 2 CTC in 40 (3.8%) samples, 3 CTC in 11 (1.0%) samples, 4 CTC in 9 (0.8%) samples and 5 or more CTC in 10 (0.9%) samples (Figure 4).

The CTC positivity rate after CHT was significantly lower when assessed using MICC (16.6%) as compared to the CTC positivity rate after CHT assessed using CSS (22.5%, *P* < 0.001). Accordingly, the distributions of CTC numbers as detected after CHT using CSS or MICC were significantly different (*P* = 0.005; see Figure 4).

In contrast to CTC positivity before CHT, CTC positivity after CHT as assessed using CSS was not significantly associated with nodal stage (*P* = 0.107). However, contrary to the situation before CHT, a significant association was found between CTC positivity after CHT determined with CSS and HER2 status of the primary tumor (*P* = 0.044), with a higher proportion of HER2 positive tumors among the CTC positive samples (105 out of 252; 41.7%) as compared to the CTC negative samples (293 out of 921; 31.8%). There were no significant associations between CTC positivity after CHT as assessed by CSS and any of the other clinico-pathological parameters listed in Table 2 (all *P* > 0.1).

Similar to samples with CTC positivity being assessed using CSS, there was a significant association between CTC positivity after CHT as assessed using MICC and the HER2 status of the tumor (*P* = 0.048), with a higher proportion of HER2 positive tumors among the CTC positive samples (48 out of 123; 39.0%) as compared to the CTC negative samples (185 out of 688; 26.9%). No other significant associations between CTC positivity after CHT as assessed using MICC and clinico-pathological parameters listed in Table 2 were found (all *P* > 0.1).

### 3.3. CTC Prevalence before and after Chemotherapy

**Figure 5:**
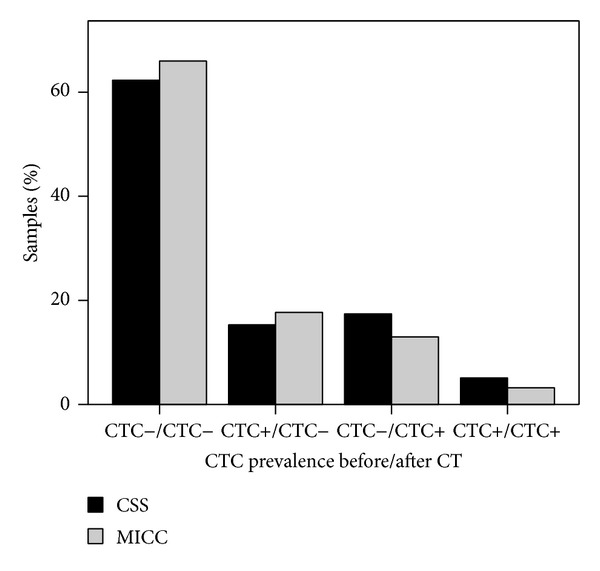
CTC prevalence before and after chemotherapy for patients whose blood samples were analyzed for the presence of CTC using either the CellSearch System (CSS, black bars; *n* = 1481) or manual immunocytochemistry (MICC, gray bars; *n* = 744) at both time points.

In 2225 patients blood samples were analyzed for the presence of CTC using the same method before and after CHT (CSS: 1481 patients; MICC: 744 patients). Regardless of the method used, most patients had no CTC in their blood at both time points (CSS: 62.3%; MICC: 66.0%) and in only a small proportion CTC were detected before and after CHT (CSS: 5.1%; MICC 3.2%). For samples analyzed using CSS the proportion of patients that were CTC negative before and CTC positive after CHT was higher as compared to samples analyzed with MICC (CSS: 17.4%; MICC: 13.0%). In contrast, the proportion of patients with CTC before but no CTC after CHT was lower in the CSS group (CSS: 15.3%; MICC: 17.7%).  Figure 5 shows the proportions of patients in the four possible categories regarding CTC presence or absence before and after CHT (i.e., CTC negative both before and after CHT; CTC negative before and CTC positive after CHT, CTC positive before and CTC negative after CHT, CTC positive both before and after CHT) when the CTC analyses at both time points were performed with either CSS or MICC. The proportions of patients in the four categories differed significantly between the two methods (*P* = 0.006).

## 4. Discussion

We evaluated CTC prevalence in peripheral blood of patients with early BC treated within the SUCCESS A trial before and after CHT in two different but comparable and well-balanced patient cohorts of the entire study-population using two different methods for CTC detection. We found ≥1 CTC/30 mL blood in 416 (21.1%) patients before and in 359 (22.5%) patients after CHT using the semi-automated CSS. Using the MICC we detected ≥1 CTC/2 × 10^6^ mononuclear blood cells (tow cytospins) in 247 (20.6%) patients before and in 177 (16.6%) patients after CHT. CTC positivity rate before CHT as assessed based on CSS was associated with a positive lymph-node status, while CTC positivity rate as assessed using MICC was not associated with any of the investigated clinical parameters. CTC positivity rate after CHT was associated with a positive HER2 status both for samples analyzed with CSS and MICC.

The CTC positivity rate as determined based on the two different methods was very similar before CHT (21.1% versus 20.6%). In contrast, the CTC positivity rate after CHT was considerably and significantly higher in samples analyzed using CSS as compared to samples analyzed using MICC (22.5% versus 16.6%).

Overall, detection rates for CTC in peripheral blood were reported in the range from 0.6% to 100% [19, 21–27]. This immense variability may be due to the broad diversity of detection methods which often lack a specific standardization and quality control. More recent data obtained from patients with metastatic BC using both immunocytochemical [1, 4, 22, 25, 28] and molecular techniques [4, 29] suggest a positivity range of 30% to 50% for a CTC positivity cut-off ≥5, and 65% to 85% for a CTC positivity cut-off ≥1 [30–33].

Only limited data exist on CTC prevalence in the adjuvant setting or during follow up period. However, compared to the metastatic situation these studies indicate an even lower prevalence of about 24% to 38% (cut-off ≥1 CTC with CSS) making the detection of CTC even more difficult [6, 34]. In patients with stage I or II BC only about 10% have ≥1 CTC/23 mL blood as stated by Wicha and Hayes [35]. Our results obtained with two different methods (CTC positivity rates of 21.1% by CSS and 20.6% by MICC before CHT, less than 10% of the patients having more than 1 CTC) are very similar to these reported values.

In our study we found comparable positivity rates before but not after CHT using CSS or MICC respectively. One possible explanation for the higher CTC positivity rate after CHT comparing CSS with MICC might be the detection of dormant cells which might not be affected by CHT. Furthermore, Aktas et al. propose that the persistence of CTC might be associated with stem cell like tumor cells and that these cells may undergo phenotypic changes, described as EMT, which enables them to escape conventional CHT [29]. This is however contradicting to the assumption, that these cells might not be detected by CSS (see below). Another hypothesis could be that CTC are somehow affected by CHT and as cell aggregates or smaller cells are lost using the OncoQuick enrichment method. Further investigations are needed to proof or neglect these theories.

A huge variety of different analytical systems for CTC isolation and detection has been developed in recent years, and attempts have been made to standardize preparation protocols and to increase assay efficiency. Two steps (isolation-enrichment and detection) are combined in most cases to identify CTC. Most of them include a separation step based on size (density gradient or filters) or biological characteristics (expression of epithelial- or cancer-specific markers), followed by the detection using ICC or molecular assays [36].

The most reliable and clinical significant results have been so far obtained using CSS as CTC detection method, which was one of the methods we evaluated. CSS is a semi-automated detection device, which importantly minimizes the source of technical failures and therefore reduces technical variability in comparison to a manual method. It is based on a combination of ICC and IF where specific markers for CTC, such as CK and EpCAM are linked to nuclear and leukocyte staining CSS has been validated in a broad clinical testing program. So far this technology has produced the largest amount of clinical data regarding CTC in BC. Different ring experiments with CSS showed very good and comparable results in the participating centers [14, 37]. In addition the supplier offers an online training every 4 months to evaluate its own results. In conclusion, CSS looks like a more reliable method compared to others because of the high standardization, the CD45 counterstaining and clearly defined selection criteria.

Nevertheless there are also some restrictions associated with the system: it is an enrichment and imaging method only, non-offering the opportunity to further characterize the cells on a molecular level. Moreover, the number of markers available per run is limited to DAPI, CK, CD45, leaving only one additional channel free for one additional marker (usually HER2). Another main disadvantage is the cell enrichment based on EpCAM only. Not all CTC express the same cell-surface antigens (such as EpCAM) and a significant subpopulation of CTC shows epithelial-mesenchymal transition (EMT)/cell stem cell (CSC) traits. Therefore enrichment methods based on EpCAM may miss these cells and underestimate the number of CTC [38–40].

Concerning MICC, several alternative protocols, different in fixatives, buffers, incubation times and antibodies, have been proposed and used [16]. In our study in order to standardize the detection of tumor cells by ICC we followed the protocol published by Fehm et al. [41]. It is important to mention that in order to save time and reduce costs, only two slides per patient were analyzed. Therefore it might be that CTC have been missed and the total CTC load has been underestimated: this may explain the lower cell numbers found using MICC compared to CSS (range before CHT: 1–256 versus 1–827, range after CHT: 1–23 versus 1–124). These differences though were not significant, since the majority of the samples contain only a low CTC load (median 1 CTC) as detected with one or the other method. However it is also possible to get false positive results since the MICC is lacking a CD45 counterstaining for leucocytes. The detection itself is quite simple though and gives no room for doubtful results, since the stained cells appear bright red.

Further characterization of CTC by use of classic ICC techniques is possible according to the investigators preference. Visual observation of stained CK-positive epithelial CTC and quantification of the staining for every single cell for different markers can provide new insights into tumor biology. Thus, MICC opens a variety of possibilities to phenotype CTC and to correlate these results with the morphology of the cells. Detecting the expression of predictive markers such as HER2, ER and other markers simultaneously [42–44] as well as deregulated pathways such as the PI3K/AKT-kinase pathway or phosphorylated EGFR to phenotype CTC further by using MICC are promising research fields [45]. These steps are required as the utilization of biomarkers for BC treatment is evolving with a high pace [46–48]. In addition, the confirmation of EMT/stem cell marker expression such as CD133 or Twist and vimentin in CTC may proof the theory that a subpopulation of CTC shows stem cell characteristics and may play a key role in the metastatic process [49].

PCR based methods, image-based approaches, microfilter and microchip devices are new technical improvements in CTC detection and characterization. The AdnaTest BreastCancer (Adna-Gen AG, Langenhagen, Germany), which is a PCR based CTC analysis method, shows an equivalent sensitivity to CSS detecting ≥2 CTC [9]. Using AdnaTest it is possible to detect very low numbers of CTC by detecting the expression of tumor associated genes, which is one advantage compared to CSS [36]. Further AdnaTest allows the detection of different additional markers such as ER, PR and EMT- or CSC-markers [50, 51] offering the possibility to further characterize the biology and molecular abnormalities of CTC to better understand the metastasizing process as well as to conduct personalized CTC directed cancer treatment [9]. However, AdnaTest does not allow a correlation with the cell morphology or with the number of cells. Since an immediate proceeding of the samples is required, it is no approach suitable for a multicenter setting.

Limitations of our study are that we evaluated two very different CTC detection methods, which differ with respect to the enrichment, the identification and the blood volume used. Further we compared the two methods in two different non-overlapping patient populations of the entire patient collective treated within the SUCCESS A trial. However the analyzes was done with very large patient numbers (*n* = 3170 before CHT and *n* = 2664 after CHT) and the patient cohorts were well-balanced concerning the common clinico-pathological parameters.

## 5. Conclusion

The detection of CTC in BC is a research field of high clinical interest and impact. The prognostic relevance in the metastatic setting is well demonstrated. Data in the adjuvant situation however is still limited. CTC detection in early BC is impaired by low positivity rate and low cell numbers. There exist a huge variety of detection methods with different advantages and disadvantages. In our study we evaluated the semi-automated CSS and MICC and found comparable CTC positivity rates before but not after adjuvant CHT in two different not overlapping but well-balanced cohorts of patient with early BC. Currently the CSS is regarded as the gold standard for CTC detection in studies aiming at investigating the prognostic value of CTC and should be further evaluated in clinical trials. However, characterization and phenotyping of CTC is crucial to deepen our understanding of metastases formation. Open systems as MICC used in our study offer the possibility to phenotype CTC and compare the marker expression with the cell morphology. In conclusion, the different approaches for CTC detection complement each other and may provide different insights in tumor biology. Highly standardized preparation protocols and high assay efficiency are strongly needed to reliably detect CTC [36].

## Figures and Tables

**Table 1:** Baseline characteristics of patients for whom CTC detection before adjuvant chemotherapy was performed using the Cell Search System (CSS) or using manual immunocytochemistry (MICC).

Variable	CSS *N* = 1972	MICC *N* = 1198	*P*-value^1^
Age (years)			0.929^2^
Median	53.0	53.0	
Range	21–78	22–85	
Tumor stage			0.707^3^
pT1	818 (41.5%)	473 (39.5%)	
pT2	1021 (51.8%)	640 (53.4%)	
pT3	100 (5.1%)	66 (5.5%)	
pT4	27 (1.4%)	16 (1.3%)	
unknown	6 (0.3%)	3 (0.3%)	
Nodal stage			0.895^3^
pN0	664 (33.7%)	418 (34.9%)	
pN1	908 (46.0%)	545 (45.5%)	
pN2	277 (14.0%)	165 (13.8%)	
pN3	123 (6.2%)	70 (5.8%)	
Histological grading			0.457^3^
G1	97 (4.9%)	59 (4.9%)	
G2	931 (47.2%)	592 (49.4%)	
G3	940 (47.7%)	544 (45.4%)	
unknown	4 (0.2%)	3 (0.3%)	
Histological type			0.170^3^
ductal	1590 (80.6%)	997 (83.2%)	
lobular	238 (12.1%)	121 (10.1%)	
other	140 (7.1%)	78 (6.5%)	
unknown	4 (0.2%)	2 (0.2%)	
Estrogen receptor status			0.697^3^
negative	589 (29.9%)	365 (30.5%)	
positive	1380 (70.0%)	829 (69.2%)	
unknown	3 (0.2%)	4 (0.3%)	
Progesterone receptor status			0.174^3^
negative	678 (34.4%)	440 (36.7%)	
positive	1289 (65.4%)	754 (62.9%)	
unknown	5 (0.3%)	4 (0.3%)	
HER2 status			0.819^3^
negative	1452 (73.6%)	886 (74.0%)	
positive	483 (24.5%)	289 (24.1%)	
unknown	37 (1.9%)	23 (1.9%)	
Menopausal status			0.560^3^
premenopausal	822 (41.7%)	512 (42.7%)	
postmenopausal	1150 (58.3%)	686 (57.3%)	
Type of surgery			0.407^3^
breast conserving	1382 (70.1%)	856 (71.5%)	
mastectomy	587 (29.8%)	340 (28.4%)	
unknown	3 (0.2%)	2 (0.2%)	
Adjuvant chemotherapy			0.930^3^
FEC-DG	968 (49.1%)	590 (49.2%)	
FEC-DOC	1004 (50.9%)	608 (50.8%)	

^1^All tests without unknowns.

^2^Mann-Whitney *U* test.

^3^Chi-square test.

FEC-DG: 3 cycles of fluorouracil-epirubicin-cyclophosphamide followed by 3 cycles of docetaxel and gemcitabine; FEC-DOC: 3 cycles of fluorouracil-epirubicin-cyclophosphamide followed by 3 cycles of docetaxel.

**Table 2:** Baseline characteristics of patients for whom CTC detection after adjuvant chemotherapy was performed using the Cell Search System (CSS) or using manual immunocytochemistry (MICC).

Variable	CSS *N* = 1598	MICC *N* = 1066	*P*-value^1^
Age (years)			0.875^2^
Median	53.0	53.0	
Range	21–76	22–79	
Tumor stage			0.842^3^
pT1	660 (41.3%)	440 (41.3%)	
pT2	824 (51.6%)	549 (51.5%)	
pT3	86 (5.4%)	62 (5.8%)	
pT4	22 (1.4%)	11 (1.0%)	
unknown	6 (0.4%)	4 (0.4%)	
Nodal stage			0.561^3^
pN0	544 (34.0%)	383 (35.9%)	
pN1	731 (45.7%)	475 (44.6%)	
pN2	221 (13.8%)	151 (14.2%)	
pN3	102 (6.4%)	57 (5.3%)	
Histological grading			0.691^3^
G1	72 (4.5%)	48 (4.5%)	
G2	770 (48.2%)	531 (49.8%)	
G3	751 (47.0%)	483 (45.3%)	
unknown	5 (0.3%)	4 (0.4%)	
Histological type			0.919^3^
ductal	1290 (80.7%)	864 (81.1%)	
lobular	191 (12.0%)	122 (11.4%)	
other	113 (7.1%)	77 (7.2%)	
unknown	4 (0.3%)	3 (0.3%)	
Estrogen receptor status			0.968^3^
negative	482 (30.2%)	322 (30.2%)	
positive	1113 (69.6%)	741 (69.5%)	
unknown	3 (0.2%)	3 (0.3%)	
Progesterone receptor status			0.587^3^
negative	549 (34.4%)	377 (35.4%)	
positive	1045 (65.4%)	686 (64.4%)	
unknown	4 (0.3%)	3 (0.3%)	
HER2 status			0.077^3^
negative	1173 (73.4%)	811 (76.1%)	
positive	398 (24.9%)	233 (21.9%)	
unknown	27 (1.7%)	22 (2.1%)	
Menopausal status			0.775^3^
premenopausal	688 (43.1%)	453 (42.5%)
postmenopausal	910 (56.9%)	613 (57.5%)
Type of surgery			0.647^3^
breast conserving	1134 (71.0%)	747 (70.1%)	
mastectomy	461 (28.8%)	316 (29.6%)	
unknown	3 (0.2%)	3 (0.3%)	
Adjuvant chemotherapy			0.624^3^
FEC-DG	780 (48.8%)	510 (47.8%)	
FEC-DOC	818 (51.2%)	556 (52.2%)	

^1^All tests without unknowns.

^2^Mann-Whitney *U* test.

^3^Chi-square test.

FEC-DG: 3 cycles of fluorouracil-epirubicin-cyclophosphamide followed by 3 cycles of docetaxel and gemcitabine; FEC-DOC: 3 cycles of fluorouracil-epirubicin-cyclophosphamide followed by 3 cycles of docetaxel.

## Abbreviations

BC: Breast cancer
CK: Cytokeratin
CSC: Cancer stem cells
CTC: Circulating tumor cell
DAPI: 4,6-diamidino-2-phenylindole
DFS: Disease free survival
DTC: Disseminated tumor cell
EMT: Epithelial-mesenchymal-transition
EpCAM: Epithelial cell adhesion molecule-1
ER: Estrogen receptor
FACS: Flow cytometry
ICC: Immunocytochemistry
IF: Immunofluorescence
MBC: Metastatic breast cancer
MRD: Minimal residual disease
OS: Overall survival
PFS: Progression free survival
PR: Progesterone receptor
RT-PCR: Reverse transcriptase-polymerase chain reaction.

## References

[1] Cristofanilli M (2006). Circulating tumor cells, disease progression, and survival in metastatic breast cancer. *Seminars in Oncology*.

[2] Pierga J-Y, Bidard F-C, Mathiot C (2008). Circulating tumor cell detection predicts early metastatic relapse after neoadjuvant chemotherapy in large operable and locally advanced breast cancer in a phase II randomized trial. *Clinical Cancer Research*.

[3] Zhang L, Riethdorf S, Wu G (2012). Meta-analysis of the prognostic value of circulating tumor cells in breast Cancer cells in breast cancer. *Clinical Cancer Research*.

[4] Muller V, Rack B, Janni W, Fasching P, Solomayer E (2012). Prognostic impact of circulating tumor cells assessed with the CellSearch System and AdnaTest Breast in metastatic breast cancer patients: the DETECT study.

[5] Rack B, Schindlbeck C, Andergassen U Prognostic relevance of circulating tumor cells in the peripheral blood of primary breast cancer patients.

[6] Lucci A, Hall CS, Lodhi AK (2012). Circulating tumour cells in non-metastatic breast cancer: a prospective study. *The Lancet Oncology*.

[7] Ross AA, Cooper BW, Lazarus HM (1993). Detection and viability of tumor cells in peripheral blood stem cell collections from breast cancer patients using immunocytochemical and clonogenic assay techniques. *Blood*.

[8] Riethdorf S, Pantel K (2008). Disseminated tumor cells in bone marrow and circulating tumor cells in blood of breast cancer patients: current state of detection and characterization. *Pathobiology*.

[9] Andreopoulou E, Yang L-Y, Rangel KM (2012). Comparison of assay methods for detection of circulating tumor cells in metastatic breast cancer: AdnaGen AdnaTest BreastCancer Select/Detect versus Veridex CellSearch system. *International Journal of Cancer*.

[10] Aurilio G, Sciandivasci A, Munzone E (2012). Prognostic value of circulating tumor cells in primary and metastatic breast cancer. *Expert Review of Anticancer Therapy*.

[11] Hayes DF, Cristofanilli M, Budd GT (2006). Circulating tumor cells at each follow-up time point during therapy of metastatic breast cancer patients predict progression-free and overall survival. *Clinical Cancer Research: An Official Journal of the American Association for Cancer Research*.

[12] Budd GT, Cristofanilli M, Ellis MJ (2006). Circulating tumor cells versus imaging—predicting overall survival in metastatic breast cancer. *Clinical Cancer Research*.

[13] Andergassen U, Kasprowicz NS, Hepp P (2013). Participation in the SUCCESS-A trial improves intensity and quality of care for patients with primary breast cancer. *Geburtshilfe Frauenheilkd*.

[14] Riethdorf S, Fritsche H, Müller V (2007). Detection of circulating tumor cells in peripheral blood of patients with metastatic breast cancer: a validation study of the CellSearch system. *Cancer Research*.

[15] Allard WJ, Matera J, Miller MC (2004). Tumor cells circulate in the peripheral blood of all major carcinomas but not in healthy subjects or patients with nonmalignant diseases. *Clinical Cancer Research*.

[16] Fehm T, Braun S, Muller V (2006). A concept for the standardized detection of disseminated tumor cells in bone marrow from patients with primary breast cancer and its clinical implementation. *Cancer*.

[17] Fehm T, Krawczyk N, Solomayer E-F (2008). ERalpha-status of disseminated tumour cells in bone marrow of primary breast cancer patients. *Breast Cancer Research*.

[18] Stigbrand T, Andres’ C, Bellangcr L (1998). Epitope specificity of 30 monoclonal antibodies against cytokeratin antigens: the ISOBM TD5-1 workshop. *Tumor Biology*.

[19] Méhes G, Witt A, Kubista E, Ambros PF (2001). Circulating breast cancer cells rre frequently apoptotic. *American Journal of Pathology*.

[20] Balic M, Dandachi N, Hofmann G (2005). Comparison of two methods for enumerating circulating tumor cells in carcinoma patients. *Cytometry B: Clinical Cytometry*.

[21] Franklin WA, Glaspy J, Pflaumer SM (1999). Incidence of tumor-cell contamination in leukapheresis products of breast cancer patients mobilized with stem cell factor and granulocyte colony-stimulating factor (G-CSF) or with G-CSF alone. *Blood*.

[22] Smith BM, Slade MJ, English J (2000). Response of circulating tumor cells to systemic therapy in patients with metastatic breast cancer: comparison of quantitative polymerase chain reaction and immunocytochemical techniques. *Journal of Clinical Oncology*.

[23] Krüger WH, Kröger N, Tögel F (2001). Disseminated breast cancer cells prior to and after high-dose therapy. *Journal of Hematotherapy and Stem Cell Research*.

[24] Krag DN, Ashikaga T, Moss TJ (1999). Breast cancer cells in the blood: a pilot study. *Breast Journal*.

[25] Kim SJ, Ikeda N, Shiba E, Takamura Y, Noguchi S (2001). Detection of breast cancer micrametastases in peripheral blood using immunomagnetic separation and immunocytochemistry. *Breast Cancer*.

[26] Brugger W, Bross KJ, Glatt M, Weber F, Mertelsmann R, Kanz L (1994). Mobilization of tumor cells and hematopoietic progenitor cells into peripheral blood of patients with solid tumors. *Blood*.

[27] Franklin WA, Shpall EJ, Archer P (1996). Immunocytochemical detection of breast cancer cells in marrow and peripheral blood of patients undergoing high dose chemotherapy with autologous stem cell support. *Breast Cancer Research and Treatment*.

[28] Pierga J-Y, Bonneton C, Vincent-Salomon A (2004). Clinical significance of immunocytochemical detection of tumor cells using digital microscopy in peripheral blood and bone marrow of breast cancer patients. *Clinical Cancer Research*.

[29] Aktas B, Tewes M, Fehm T, Hauch S, Kimmig R, Kasimir-Bauer S (2009). Stem cell and epithelial-mesenchymal transition markers are frequently overexpressed in circulating tumor cells of metastatic breast cancer patients. *Breast Cancer Research*.

[30] Fehm T, Sauerbrei W (2010). Information from CTC measurements for metastatic breast cancer prognosis-we should do more than selecting an ‘optimal cut point’. *Breast Cancer Research and Treatment*.

[31] Bidard F-C, Mathiot C, Degeorges A (2010). Clinical value of circulating endothelial cells and circulating tumor cells in metastatic breast cancer patients treated first line with bevacizumab and chemotherapy. *Annals of Oncology*.

[32] Botteri E, Sandri MT, Bagnardi V (2010). Modeling the relationship between circulating tumour cells number and prognosis of metastatic breast cancer. *Breast Cancer Research and Treatment*.

[33] Pierga J-Y, Hajage D, Bachelot T (2012). High independent prognostic and predictive value of circulating tumor cells compared with serum tumor markers in a large prospective trial in first-line chemotherapy for metastatic breast cancer patients. *Annals of Oncology*.

[34] Lang JE, Mosalpuria K, Cristofanilli M (2009). HER2 status predicts the presence of circulating tumor cells in patients with operable breast cancer. *Breast Cancer Research and Treatment*.

[35] Wicha MS, Hayes DF (2011). Circulating tumor cells: not all detected cells are bad and not all bad cells are detected. *Journal of Clinical Oncology*.

[36] Lianidou ES, Markou A (2011). Circulating tumor cells in breast cancer: detection systems, molecular characterization, and future challenges. *Clinical Chemistry*.

[37] Kraan J, Sleijfer S, Strijbos MH (2011). External quality assurance of circulating tumor cell enumeration using the CellSearch V system: a feasibility study. *Clinical Cytometry*.

[38] Sieuwerts AM, Kraan J, Bolt J (2008). Anti-epithelial cell adhesion molecule antibodies and the detection of circulating normal-like breast tumor cells. *Journal of the National Cancer Institute*.

[39] Fehm T, Solomayer EF, Meng S (2005). Methods for isolating circulating epithelial cells and criteria for their classification as carcinoma cells. *Cytotherapy*.

[40] Königsberg R, Obermayr E, Bises G (2011). Detection of EpCAM positive and negative circulating tumor cells in metastatic breast cancer patients. *Acta Oncologica*.

[41] Fehm T, Braun S, Muller V (2006). A concept for the standardized detection of disseminated tumor patients with primary breast. *Cancer*.

[42] Bock C, Rack B, Kuhn C (2012). Heterogeneity of ER*α* and ErbB2 status in cell lines and circulating tumor cells of metastatic breast cancer patients. *Translational Oncology*.

[43] Rack B, Bock C, Andergassen U, Doisneau-Sixou S (2012). Hormone receptor status, erbB2 expression and cancer stem cell characteristics of circulating tumor cells in breast cancer patients. *Histology and Histopathology*.

[44] Andergassen U, Zebisch M, Kolbl AC (2012). Immuno-histochemical evidence of disseminated tumor cells from the bone marrow of female breast cancer patients: correlation of Her2 and thomsen-friedenreich-antigen. *Geburtsh Frauenheilk*.

[45] Kallergi G, Agelaki S, Kalykaki A, Stournaras C, Mavroudis D, Georgoulias V (2008). Phosphorylated EGFR and PI3K/Akt signaling kinases are expressed in circulating tumor cells of breast cancer patients. *Breast Cancer Research*.

[46] Schmidt M, Fasching PA, Beckmann MW (2012). Biomarkers in breast cancer—an update. *Geburtshilfe und Frauenheilkunde*.

[47] Luftner D, Lux MP, Maass N (2012). Advances in breast cancer—looking back over the year. *Geburtshilfe und Frauenheilkunde*.

[48] Melcher C, Scholz C, Jäger B, Hagenbeck C, Rack B, Janni W (2012). Breast cancer: state of the art and new findings. *Geburtshilfe und Frauenheilkunde*.

[49] Kallergi G, Papadaki MA, Politaki E, Mavroudis D, Georgoulias V, Agelaki S (2011). Epithelial to mesenchymal transition markers expressed in circulating tumour cells of early and metastatic breast cancer patients. *Breast Cancer Research*.

[50] Kasimir-Bauer S, Hoffmann O, Wallwiener D, Kimmig R, Fehm T (2012). Expression of stem cell and epithelial-mesenchymal transition markers in primary breast cancer patients with circulating tumor cells. *Breast Cancer Research*.

[51] Aktas B, Müller V, Tewes M (2011). Comparison of estrogen and progesterone receptor status of circulating tumor cells and the primary tumor in metastatic breast cancer patients. *Gynecologic Oncology*.

